# Trichophyton species isolated from asymptomatic patients of the pet-owner pair in Mexico

**DOI:** 10.18502/cmm.7.2.7029

**Published:** 2021-06

**Authors:** Roberto Adame- Gomez, Monica Gisela Rodrigez- Romero, Isabel Hilario- Alejandro, Sandra Alheli Pineda- Rodriguez, Jeiry Toribio- Jimenez, Elvia Rodriguez- Bataz, Arturo Ramirez- Peralta

**Affiliations:** 1 Microbial Pathometabolism Research Laboratory, The Autonomous University of Guerrero, Chilpancingo, México; 2 Parasitology Research Laboratory, The Autonomous University of Guerrero, Chilpancingo, México; 3 Molecular Microbiology and Environmental Biotechnology Laboratory, The Autonomous University of Guerrero, Chilpancingo, México

**Keywords:** México, Mycoses, Pet, Pet owners

## Abstract

**Background and Purpose:**

Superficial mycoses are the fourth most common cause of disease worldwide. It is not surprising that zoonotic transmission occurs to humans due to close contact with different animals, be it companion or farm animals. Therefore, the objective of this study was to determine the presence of asymptomatic dermatophyte carriers in the owner-pet pairs, identify the most common etiologic agents, and find the likely connection between the carrier status of an owner and the presence of dermatophytes in their pets.

**Materials and Methods:**

From May 2019 to January 2020, 21 cats and 115 dogs with their respective owners were selected for dermatophyte culture. All the dogs and cats included in the study were from the communities of southeastern Mexico. The samples were taken with a cotton swab, which was vigorously rubbed and twisted on the scalp or body of the pet four times and grown on Mycosel Agar. The isolates were identified based on macroscopic and microscopic characteristics. The prevalence of the binomial ranged from 0.73% in pet skin and human hands to 2.2% in human scalp. In humans, the agents were *Trichophyton tonsurans* and *Trichophyton verrucosum*, while in pets, a strain of *Trichophyton sp* was found.

**Conclusion:**

Different species of dermatophytes were found in the owner/pet pairs, which denotes that coexistence is not related in asymptomatic cases.

## Introduction

Dermatophytes are the most common cause of superficial fungal infections. They are a specialized group of filamentous fungi capable of infecting and degrading keratinized tissues, including skin, hair, and nail [ 1 ]. Every year, millions of people are affected by these infections worldwide [ 2 ] which cost hundreds of millions of dollars due to medications (estimates that do not consider the additional cost of medical visits and loss of time in work and school) [ 3 ]. 

It is not surprising that zoonotic transmission occurs to humans due to close contact with either companion or farm animals. In this regard, Cafarchia et al. have described the transmission of *Microsporum canis* from dogs and cats to their owners [ 4 ] and the presence of arthrospores in the environment in which they live [ 5 ]; however, the owners may not have symptoms.

Therefore, this study aimed to determine the presence of asymptomatic dermatophyte carriers in the owner-pet pairs, identify the most common etiologic agents, and find the likely connection between the carrier status of an owner and the presence of dermatophytes in their pets, whether feline or canine.

## Materials and Methods

From May 2019 to January 2020, 21 cats and 115 dogs with their respective owners were selected for dermatophyte culture. All the dogs and cats included in the study were from the communities of Chilpancingo, Acalmani, and Chilapa (Guerrero, southeastern Mexico) and were examined at their respective homes. The pairs lived in urban (Chilpancingo), suburban (Chilapa), and rural (Acalmani) areas. It should be mentioned that only pairs, meaning one animal one human per house, were included in this study. Furthermore, the inclusion criteria were lack of skin lesions and no history of dermatophytosis during the months before sampling.

After the informed consent was obtained from the pet owners, the socio-demographic and clinical characteristics of the pairs were collected via a questionnaire. Regarding ethical considerations, the information of patients was handled according to the Helsinki declaration. The informed consent and the research were approved by the Ethics Committee of the Universidad Autonoma de Guerrero (FCQB200619). 

Each pair was examined for detached hair and/or alopecia, peeling, and crusting. However, samples of the scalp or body of the pets (defined as both animal fur and fur) were taken of all pairs, regardless of whether they had clinical symptoms or not. The samples were taken with a cotton swab, which was vigorously rubbed and twisted on the scalp or body of the pets four times. 

After the procedure was completed, the cotton swab was placed in 0.1% Tween. Since all the patients in the study were asymptomatic, we considered using this method as it is the least invasive and has the same reliability as the toothbrush method [ 6 ]. Fungal cultures were performed by inoculations of the samples in Petri dishes containing Mycosel agar (manufactured by BD, USA) and incubated at 28 °C for 15 days. Colonial growth in the medium was identified by species, based on its morphology and microscopic characteristics of the hyphae, macroconidia, and microconidia. If necessary, it was sub-cultured on potato dextrose agar (manufactured by BD, USA). 

A database was created with the information collected from the questionnaires and the laboratory results. The frequency analysis was carried out using the Xi-square statistical test and the values of *P*<0.05 were considered statistically significant.

## Results and Discussion

In total, 136 pet-owner pairs were sampled, and dermatophytes were found in 3.67% of them which reflected a total of five strains, three of which were isolated in Chilpancingo, one in Chilapa, and one in Acalmani, without statistically significant differences. Moreover, four out of the five culture-positive cases were isolated from owners (three in scalp and one in hands) and one from a pet.

According to the macroscopic and microscopic characteristics of the isolates as well as the growth on Mycosel Agar, two of the scalp isolates were identified as *Trichophyton tonsurans* and one as *T. verrucosum*. In the micro-morphology of the two remained isolates from the skin of one owner and one pet, thin hyphae and microconidia along with hyphae were identified microscopically. However, other fungal characteristic structures were not identified and thereby two isolates were reported as *Trichophyton sp*. In this sense, several dermatophytes are known which do not or poorly sporulate in culture and thereby show very limited phenotypic characteristics [ 7 ]. 

Regarding the characteristics of the pets, the majority of them were dogs with 81.69%, 82.5%, and 96% in Chilpancingo, Chilapa, and Amalcami respectively. As for family pet-owner coexistence, patients shared their pillows with their pets more frequently in Chilpancingo (88.3%), compared to the other locations (65% in Chilapa and 68% in Acalmani) (*P*=0.010). Regarding their behaviors as pairs, in Chilpancingo, 18.31% of owners and pets always slept together, while in Chilapa, 92.5% of the pairs never did (*P*=0.001). Moreover, 38.03%, 12.5%, and 12% of the pet owners in Chilpancingo, Chilapa, and Acalmani always fed their pets, respectively (*P*=0.001). It was also observed that 78.8%, 95%, and 32% of the pets in Chilpancingo, Chilapa, Acalmani were not taken to public parks, respectively (*P*=0.001) (Table 1).The prevalence of the pairs was 3.67% based on the sample type and ranged from 0.73% in the skin of pets and owners to 2.2% in the scalp of the owners. In this study, it was decided to take a sample from these parts of the body in pet owners due to their common contact with pets, both at the time of coexistence and while practicing hygiene habits.

**Table 1:** Data of pet-owners

Variable	Chilpancingo N=71 %(n/N)	Chilapa de Álvarez N=40 %(n/N)	Acalmani N=25 %(n/N)	*P*
Pet characteristics				
**Family**				0.214
Canidae	81.69 (58/71)	82.50(33/40)	96.00 (24/25)	
Felidae	18.31 (13/71)	17.50(7/40)	4.00 (1/25)	
Gender				
Male	45.07 (32/71)	50.00 (20/40)	44.00 (11/25)	0.854
Female	54.93 (39/71)	50.00 (20/40)	56.00 (14/25)	
Family environment of the pet owners				
**Pillow sharing**				0.01
Never	5.63 (4/71)	30.00 (12/40)	12.00 (3/25)	
Rarely	1.41 (1/71)	0.00 (0/40)	8.00 (2/25)	
Occasionally	1.41 (1/71)	0.00 (0/40)	4.00 (1/25)	
Often	4.23 (3/71)	5.00 (2/40)	8.00 (2/25)	
Always	87.32 (62/71)	65.00 (26/40)	68.00 (17/25)	
Pet-owner interaction				
**Sleeping with pets**				
Never	54.93 (39/71)	92.50 (37/40)	36.00 (9/25)	0.001
Rarely	11.27 (8/71)	7.50 (3/40)	48.00 (12/25)	
Occasionally	4.23 (3/71)	0.00 (0/40)	8.00 (2/25)	
Often	11.27 (8/71)	0.00 (0/40)	8.00 (2/25)	
Always	18.31 (13/71)	0.00 (0/40)	0.00 (0/25)	
**Pet food intake**				
Never	19.72 (14/71)	75.00 (30/40)	48.00 (12/25)	0.001
Rarely	7.04 (5/71)	7.50 (3/40)	4.00 (1/25)	
Occasionally	18.31 (13/71)	2.50 (1/40)	24.00 (6/25)	
Often	16.90 (12/71)	2.50 (1/40)	12.00 (3/25)	
Every day	38.03 (27/71)	12.50 (5/40)	12.00 (3/25)	
**Visits to parks**				
Never	78.87 (56/71)	95.00 (38/40)	32.00 (8/125)	0.001
Rarely	11.27 (8/71)	0.00 (0/40)	48.00 (12/25)	
Occasionally	1.41 (1/71)	2.50 (1/40)	8.00 (2/25)	
Often	5.63 (4/71)	0.00 (0/40)	0.00 (0/25)	
Every day	2.82 (2/71)	2.50 (1/40)	12.00 (3/25)	

Even though the same dermatophytes were not found on the skin of pets and the scalp and hands of pet owners, it is important to describe the collected data. In a study performed by Cafarchia et al., the prevalence rates of dermatophytes in cats and dogs were found to be 28.2% and 20.5% respectively, while in our investigation this prevalence rate was lower. The likely explanation for such a significant difference could be the low number of sampled cats (n=21) in the present study. It could also be due to the inclusion of two groups in the study conducted by Cafarchia et al. [ 4 ], in one of which, the owners were diagnosed with dermatophytosis, while in the present study, all the pets were asymptomatic. 

In addition, the prevalence is commonly high in owner-pet pairs that include symptomatic cases [ 8 ]. A high presence of contaminated surfaces has even been reported in households where there are pets with signs and symptoms [ 9 ]. The low prevalence rate of dermatophytes in the present study could be explained by the lack of symptomatic cases in our population and the low number of sampled cats. It should be noted that based on previous studies, cats play an important role as asymptomatic carriers in the transmission of dermatophytes [ 10 ].

In another study, Debnath et al. found that 20.93% of healthy dogs were positive for dermatophytes; however, they described that the age of the dogs and the season of the year can influence the frequency of dermatophytes [ 11 ]. Regarding this last point, all the seasons of the year were not considered in the present research.

Regarding hand samples, there is little information available about the patients without apparent symptoms. However, such asymptomatic lesions could probably be related to the low virulence of the strains existing in these lesions, for which enzymatic profiles of dermatophytes strains recovered from these infections could even be tested as previously described (keratinases, gelatinases, proteases, and lipases) [ 12 ]. 

Likewise, the development of infection in the owner can be attributed to the characteristics of the pet-owner interaction and the duration of their contact [ 2 ]. Nevertheless, asymptomatic scalp infection by dermatophytes in humans (as asymptomatic carriers),

which plays an important role in zoonotic transmission, is well known [ 13 , 14 , 15 ]. They are commonly found in children and places with a high prevalence of tinea capitis [ 16 ]. Such prevalence in this study was within the reported range from 0.1 to 49% [ 17 , 18 ]. With an even lower prevalence rate in pets and different dermatophytes, other factors could be considered favoring the presence of dermatophytes in pet owners. 

In this study, it was found that the three studied populations had different hygiene habits and lifestyles based on their residence location, namely locality urban (Chilpancingo), peri-urban (Chilapa), and rural (Acalmani) areas. One example of this would be the sharing of pillows; while the pet always slept with the owner in 18.3% of cases in Chilpancingo, this activity was not common in other communities (*P*= 0.001). Therefore, it could not be the factor favoring the appearance of asymptomatic dermatophyte carriers. 

Even though higher percentages of owners who fed their pet with formulated pet food and had a low number of visits to the park were observed in Chilpancingo, dermatophyte was found among them. This could be related to constant pet grooming due to the close coexistence with the owner (in all the surveyed pairs the pet lived inside the house and at least 45% of the pairs slept together). 

Although the presence of dermatophytes on the scalp of apparently healthy individuals may, in some cases, be a temporary event, it is reasonable to assume that asymptomatic carriers play a role in the spread and persistence of scalp ringworm in the community [ 18 ]. These people could be the source by which dermatophytes are transferred as fungal agents to others via clothes or in public places, such as schools and gyms [ 19 , 20 ].

Regarding etiology, the presence of *T. tonsurans* was determined in asymptomatic carriers. In this sense, the etiological agent has been found as specific to the asymptomatic carriers [ 13 , 14 ]. In addition, *T. tonsurans* has been reported to have displaced almost all the species of dermatophytes that cause tinea capitis in North America [ 14 , 21 ]. This microorganism has recovered with an increased frequency in infections in European and East Asian countries [ 22 - 24 ]. Furthermore, *T. tonsurans* plays a central role in symptomatic disease and can be recovered from hosts without symptoms (asymptomatic carriers) at high rates, comparable to the rates observed in patients with clinical infection [ 20 , 25 , 26 , 27 ] which is consistent with the findings of this study. 

It should be noted that *T. verrucosum* has also been found in asymptomatic carriers of Tinea capitis and is considered an important etiologic agent [ 18 , 28 , 29 ] in addition to others, such as *M. audouinii* [ 15 ]. In contrast, zoophilic microorganisms, such as *M. canis* or *T. mentagrophytes*, are usually present with a symptomatic inflammatory response and are less frequently associated with a carrier status [ 13 , 14 , 30 ].

It is noteworthy that two slow-growing filamentous fungi were isolated from the skin of pets and scalp of a patient on Mycosel agar with short hyphae and the presence of microconidia. However, we were unable to observe other structures, such as spiral or tendril hyphae, cigar-shaped macroconidia, or colony pigmentation; hence, they were only defined as *Trichophyton sp*. 

## Conclusion

Lifestyle, which is different in various localities (populations), can help the development of carrier state in people who have notable contact with pets. Isolation of different dermatophyte species from the skin of pet/owner pairs in this study denoted an asymptomatic carrier state in humans that cannot be related to the coexistence of the same isolate in the pets. 

## Acknowledgement

The authors would like to appreciate Lilia Lizette García Díaz (biotechnology) and Alberto Patricio Hernández (chemistry) for their support in the conservation of strains and all owners and their pets. 

## Authors’ contribution

Conceptualization: A.R.P. and R.A.G; methodology: M.G.R.R. and I.H.A; supervision: A.R.P.; statistical analysis: S.A.P.R and E.R.B; investigation: R.A.G and A.R.P; writing and original draft preparation: A.R.P., R.A.G, and J.T.J. All authors read and approved the final manuscript.

## Conflict of Interest

The authors declare no conflicts of interest related to this study.

## Funding

This research was not funded by any program.

## Financial disclosure

No financial interests related to the material of this manuscript have been declared.
